# The prevalence of renal impairment in individuals seeking HIV testing in Urban Malawi

**DOI:** 10.1186/s12882-016-0403-7

**Published:** 2016-11-22

**Authors:** Nicola Glaser, Sam Phiri, Tom Bruckner, Dominic Nsona, Hannock Tweya, Nomeda Ahrenshop, Florian Neuhann

**Affiliations:** 1 Institute of Public Health, University of Heidelberg, Heidelberg, Germany; 2 The Lighthouse Trust, Lilongwe, Malawi; 3 Department of Medicine, University of North Carolina, Chapel Hill, USA; 4 Institute of Medical Biometry and Informatics, University of Heidelberg, Heidelberg, Germany; 5 International Union against Tuberculosis and Lung Disease, Paris, France

**Keywords:** Chronic kidney disease, Glomerular filtration rate, Nephrology, HIV, Non-communicable diseases, Sub-Saharan Africa, LMIC, Malawi, Schistosomiasis

## Abstract

**Background:**

Chronic kidney disease (CKD) poses a major health threat to people living in low- and middle-income countries, especially when it is combined with HIV, antiretroviral treatment (ART) or communicable and non-communicable diseases. Data about the prevalence of CKD and its association with other diseases is scarce, particularly in HIV-negative individuals. This study estimated the prevalence of CKD in individuals who were either HIV-positive (and ART-naïve) or HIV-negative in an urban Malawian population.

**Methods:**

This cross-sectional study was conducted at a HIV Testing and Counselling Centre in Lilongwe, Malawi. Consecutive clients who were ≥18 years and consented to participate were enrolled over a 3-month period. Clients were screened for potential renal disease and other conditions. Their blood pressure was measured, urine examined via dipstick and albumin/creatinine ratio and blood drawn for creatinine, cystatin C and sero-markers for schistosomiasis. Estimated glomerular filtration (eGFR) rate was calculated using a cystatin C-based formula and classified according to the matching CKD stages by K/DOQI (The National Kidney Foundation Kidney Disease Outcome Quality Initiative). We performed a descriptive analysis and compared differences between HIV-positive (and ART naïve) and -negative participants.

**Results:**

Out of 381 consecutive clients who were approached between January and March 2012, 366 consented and 363 (48% female; 32% HIV-positive) were included in the analysis. Reasons for exclusion were missing samples or previous use of ART. HIV-positive and negative clients did not differ significantly with regard to age, sex or medical history, but they did differ for BMI—21.3 (±3.4) vs. 24 (±5.1), respectively (*p* < 0.001). Participants also differed with regard to serum cystatin C levels, but not creatinine. Reduced kidney function (according to CKD stages 2–5) was significantly more frequent 15.5 vs. 3.6%, respectively (*p* < 0.001) among HIV-positive clients compared to the HIV-negative group. Differences in renal function were most pronounced in the eGFR range 60–89 ml/min/1.73 m^2^ accompanied by proteinuria with results as 11.2% vs. 1.2%, respectively for clients who were HIV-positive vs. HIV-negative (*p* = 0.001).

**Conclusions:**

Reduced glomerular filtration and/or proteinuria occurred in 15.5% of HIV-positive, and 3.6% of HIV-negative patients in this urban Malawian cohort.

Since generalized renal monitoring is not feasible in Malawi or other resource-limited countries, strategies to identify patients at risk for higher stages of CKD and appropriate preventive measures are needed for both HIV-positive and HIV-negative patients.

**Electronic supplementary material:**

The online version of this article (doi:10.1186/s12882-016-0403-7) contains supplementary material, which is available to authorized users.

## Background

Chronic kidney disease (CKD) is categorized as a non-communicable disease (NCD), a group that includes several globally prioritized diseases including diabetes, chronic respiratory disease, cancer and cardiovascular disease. NCDs account for 60% of all deaths worldwide, and 80% occur in low- and middle-income countries (LMIC) [1, 2], which underlines the assumption that NCDs are associated with poverty [3]. CKD increases the risk for developing hypertension and cardiovascular disease and also exacerbates the course of these illnesses. CKD occurs more frequently in patients with hypertension, cardiovascular disorders or diabetes [4].

The consequences of CKD are hypothesized to be worse in LMIC because of the high burden of non-communicable and communicable diseases, such as HIV infection and tuberculosis as well as infectious tropical diseases coupled with limited access to health care facilities for routine screening [5–7]. Calculations suggest that approximately 200–300 individuals per million are affected by CKD in sub-Saharan Africa (SSA) [8].

HIV is highly prevalent in SSA and constitutes an independent risk factor for kidney disease due to the direct and indirect effects of the infection, e.g., HIV-associated nephropathy (HIVAN). Diseases associated with HIV-infection (e.g., schistosomiasis or tuberculosis) can also play an important role in a patient developing CKD [9]. Additionally, drugs used in antiretroviral treatment (ART) such as tenofovir disoproxile fumarate (TDF) can affect renal function, especially in people with underlying CKD [10].

Despite a high HIV-prevalence of approximately 11% in Malawi [11] and the associated risks for CKD, current knowledge about the prevalence of CKD or renal diseases in SSA countries including Malawi is scarce and renal registries are non-existent. In the existing literature findings on the prevalence of CKD in SSA range from 4.7% in a HIV-negative cohort in Uganda [12] to 33.5% in HIV-positive people in Zambia [13]. It is not easy to determine whether these differences resulted from actual differences in prevalence, varying thresholds and CKD definitions, or if they were influenced by the inability to compare the various equations and laboratory methods used to assess kidney function in SSA [14].

This cross-sectional descriptive study estimated the prevalence of renal impairment in an adult urban Malawian population of HIV-positive (and ART naïve) and HIV–negative individuals. We also describe the prevalence of potential risk factors for CKD in HIV-positive patients before the use of TDF in first line ART.

## Methods

A cross-sectional study was performed at the HIV Testing and Counselling (HTC) centre at the Lighthouse HIV clinic (two centres) in Lilongwe, Malawi between the 24th of January 2012 and the 29th of March 2012. The Lighthouse serves a mainly urban population of approximately 670,000 people living among a larger population of 1,230,000 people in Lilongwe [15].

All clients who were ≥18 years of age and ART-naïve, who came for HTC at the Lighthouse centre during the study period were approached to enrol. After providing informed consent, a standardized questionnaire about age, gender, HIV-status, possible pregnancy, current symptoms and medical and family history was completed. Body height and weight were taken. Blood pressure (BP) was measured using a calibrated standard automatic BP device (Omron, Germany). It was used on the free right arm at heart level. Clients sat for at least 10min with their backs touching the chair backrest [16]. Hypertension was defined as history of hypertension, use of antihypertensive medication or BP values ≥ 140 systolic or ≥ 90 diastolic. Data were excluded if samples were missing or there was a history of using ART. The study methodology has been described in detail elsewhere [16]. The dataset used for this article can be found in Additional file 1.

### Laboratory measurements

Venous blood (5 ml) and a midstream-urine sample were collected. An aliquot of serum and one of urine were frozen at −80 °C within 6 hours after performing dipstick, centrifugation and microscopy. If there was a positive protein patch or a positive haemoglobin/erythrocytes patch on the dipstick, urine microscopy was performed. Analyses for serum creatinine, serum cystatin C and urine albumin-creatinine-ratio (ACR) were performed at the University of Heidelberg, Germany (samples were sent on dry ice by air). Creatinine levels in serum and urine were photometrically analysed; cystatin C and urine albumin were assessed by a turbidimetric method (ADVIA 2400 Siemens Healthcare Diagnostics) [16]. Schistosomiasis serology was performed at a reference laboratory for schistosomiasis in the Department of Clinical Tropical Medicine at the University of Heidelberg. Schistosoma-egg-based ELISA and parenchymal and focal indirect immunofluorescence assays were used. Results were classified into positive, borderline or negative according to lab standards.

### Assessment of renal function

A cystatin C-based estimated glomerular filtration rate (eGFR) was calculated using the formula of Van Deventer et al. developed in a comparable cohort in South Africa [17] and applied in the final analysis. For comparison, we also calculated eGFR with the Cockcroft-Gault equation as elaborated in Glaser et al. [16]. Renal function was classified according to K/DOQI eGFR categories for CKD [18] and kidney damage was defined as proteinuria with an albumin-creatinine ratio > 3 mg/mmol creatinine.

### Statistical analysis

Data entry was done in MS Access 2007 and analysed with STATA 10 and 11. Significance in the differences between HIV-positive and -negative participants as well asassociations were tested using a *t*-test and chi-squared test according to the underlying distribution and a Spearman’s test for correlation; the level of significance was set at *p* > 0.05 [16].

### Ethical clearance

The study protocol received clearance by the ethical committee of the University of Heidelberg and the National Health Sciences Research Committee of Malawi.

## Results

Out of 381 consecutive clients invited to participate, 366 consented and 363 (48% female) were included in the final analysis. Reasons for exclusion were missing samples or previous ART treatment. Of the included participants, 116 (32%) and 247 (68%) were HIV-positive and HIV-negative, respectively. Eight women were pregnant and 13 were breastfeeding. Ages ranged from 19 to 69 years. The mean BMI was 23.2 (SD = 4.8), but was significantly lower in HIV-positive compared to HIV-negative individuals: 21.3 (SD = 3.4) vs. 24.0 (SD = 5.10), respectively (*p* < 0.001). Mean serum cystatin C was 0.82 mg/l (SD = 0.23), and differed significantly between HIV-positive and –negative participants: 0.90 mg/l (SD = 0.18) and 0.79 mg/l (SD = 0.24) respectively (*p* < 0.001). Elevated BP was defined as ≥140 systolic or ≥90 diastolic and was found in 49 (14%) participants, (40 HIV-negative, 9 HIV-positive; *p* = 0.028). Fifteen participants (4%) reported having had an earlier diagnosis of diabetes mellitus and one participant was diagnosed with diabetes mellitus during this study (Table 1).

**Table 1:** Patient characteristics of the participants of the RESULT study conducted at the Lighthouse Clinic in urban Lilongwe, Malawi between January and March 2012

	Total Mean (SD) or N (% of total), *N* = 363	HIV+ (%), *N* = 127	HIV- (%), *N* = 247	*p* value
Age	34.1 (±10.9)	33.9 (±9.3)	34.2 (±11.6)	0.81*
Sex				
Women	174 (48%)	57 (49%)	117 (47%)	0.75**
Men	189 (52%)	59 (51%)	130 (53%)	
BMI	23.2 (±4.8)	21.3 (±3.4)	24. (±5.10)	<0.001*
Serum creatinine (mg/dl)	0.76 (±0.31)	0.74 (±0.21)	0.78 (±0.34)	0.21*
Serum cystatin C (mg/L)	0.82 (±0.23)	0.90 (±0.18)	0.79 (±0.24)	<0.001*
eGFR by cystatin C	96.39 (±15.61)	90.87 (±14.75)	98.98 (±15.36)	<0.001*
Proteinuria				
ACR > 3 mg/mmol	44 (12.1%)	24 (20.7%)	20 (8.1%)	0.001**
No	319 (87.9%)	92 (79.3%)	227 (91.9%)	
BP ≥ 140 systolic or				
≥ 90 diastolic	49 (13.5%)	9 (7.8%)	40 (16.2%)	0.028**
*No*	314 (86.5%)	107 (92.2%)	207 (83.8%)	
Schistosomiasis				
Positive (IIFT > 1:40 or ELISA OD^a^ >3)	123 (33.9%)	34 (29.3%)	89 (36%)	0.379**
Negative or borderline	240 (66.1%)	82 (70.7%)	158 (63.9%)	

*t-test; **χ ^2^ test

^a^ *IIFT* indirect immunofluorescent assay, *ELISA* enzyme-linked immunosorbent assay optical density

Urine samples from 71 patients were examined with a microscope; 21 (29.6%) had bacteriuria, 3 (4.2%) had trichomonas and 4 (5.6%) had schistosoma haematobium eggs.

Schistosoma-antibody ELISA and indirect immunofluorescent assay testing were performed and 123 participants (34%) were found to have positive serology indicating recent or past infection with either schistosoma haematobium or schistosoma mansoni.

Three participants reported having previous kidney diseases. One reported having had glomerulonephritis, while the other two could not specify their disease.

Although a total of 47 (13%) participants suffered from renal impairment, and 11 (3.1%) had moderate to severe renal dysfunction, none of those had had a previous diagnosis of kidney disease.

CKD (stages 1–5) was significantly more common in HIV-positive individuals: 15.5% of HIV-positive vs. 3.6% of HIV-negative participants (*p* < 0.001). Proteinuria and HIV were also significantly associated (*p* = 0.001) (Table 2).

**Table 2:** Participants of the RESULT study, conducted in Lilongwe Malawi and listed according to CKD stages as defined by K/DOQI [20] and HIV status

Kidney function (eGFR in ml/min/1.73 m^2^)	Total, *n* = 363 (%)	HIV-positive, *n* = 116 (%)	HIV-negative, *n* = 247 (%)
Stage 0: No impairment:			
eGFR ≥ 60, − proteinuria	316 (87)	90 (77.6)	226 (91.5)
Stage 1: Kidney damage with normal or ↑GFR:			
eGFR ≥ 90 + proteinuria	20 (5.5)	8 (6.9)	12 (4.9)
Stage 2: Kidney damage with mild ↓ GFR:			
eGFR 60–89 + proteinuria	16 (4.4)	13 (11.2)	3 (1.2)
Stage 3: Moderate ↓ GFR:			
eGFR 30–59 ± proteinuria	9 (2.5)	5 (4.3)	4 (1.6)
Stage 4: Severe ↓ GFR:			
(eGFR 15–29 ± proteinuria)	2 (0.6)	-	2 (0.8)

Higher age (age groups: 18–30, 30–45 and >45 years) and higher stages of CKD (CKD stage 2–5, (*p* < 0.001) and CKD stage 3–5, (*p* < 0.001), respectively) were associated. Testing with Spearman’s test of correlation led to the same result: CKD stage 2–5 (*p* < 0.001), rho = 0.22; CKD 3–5 (*p* = 0.001), rho = 0.21. CKD stage 3–5 was also significantly associated with male gender (*p* = 0.045) and hypertension (*p* = 0.009) (defined as systolic BP ≥ 140 or diastolic BP ≥ 90 mmHg either measured or reported). Although diabetes and higher stages of CKD showed a trend toward association, there was no statistical significance. Nor was there any significant association of CKD and schistosomiasis.

## Discussion

The general prevalence of CKD stage 2–5 in the study group was 7.5% with 3.1% suffering from moderate to severe impairment corresponding to CKD stages 3–5. We found a significant difference between HIV-positive and –negative individuals regarding reduced eGFR. HIV-status, age and hypertension were associated with CKD.

Our study found a lower prevalence of reduced eGFR in HIV-positive (15.5%) than a study conducted in Blantyre in 2009, where 57.4% of the participants with WHO clinical stage I or II for HIV [19] presented with a reduced creatinine clearance of <90 ml/min [20]. This study estimated GFR using the Cockcroft-Gault formula, which we also did in a sensitivity analysis as elaborated in Glaser et al. [16]. The difference cannot be explained by the use of various formulas alone, nor can it be explained by creatinine determination or composition of the cohorts since we did not exclude patients with more advanced stages of HIV infection. Therefore, this discrepancy in findings warrants further investigation. As expected, higher stages of CKD in our study were associated with an older age [21]. This was also true for hypertension, which is known to be one of the main risk factors for CKD [22]. Due to the small number of participants with diabetes (another established risk factor for CKD), we were unable to confirm an association with CKD.

Proteinuria was very common in HIV-positives, which is also supported by other studies [23]. Although especially heavy proteinuria [24] is known to occur in HIVAN [25], we did not often find HIVAN in the HIV-positive group.

The prevalence of serological markers for past or recent schistosomiasis infection was higher than expected among this adult study cohort. Despite the fact that schistosomiasis is a known risk factor for developing CKD [26] or acquiring HIV [27], we did not find a significant association in either case.

Levels of elevated BP were found to be much lower (13.5%) in this study than in the Malawi STEP survey of 2009 where elevated BP (diastolic BP ≥ 90 mmHg or systolic ≥ 140 mmHg or on medication) was found in 32.9% [28]. This difference in BP levels warrants further investigation since intuitively one would assume that HIV testing leads to higher BP levels.

The study has several limitations. Due to limited laboratory facilities and the study protocol, we did not use an acknowledged gold-standard method to assess GFR. After studying the literature, we decided to use cystatin C measurement as a quasi-gold standard, because is it less dependent on muscle mass and appears to show the best results in HIV-positive individuals [29] including those in SSA [17]. At the time of this study, the certified reference material developed by the International Federation of Clinical Chemistry and Laboratory Medicine (IFCC) and recommended by KDIGO in 2012 [30], had not yet been introduced and therefore, was not used. However, analysis was performed at the University of Heidelberg laboratory, thus fulfilling the highest quality control standards.

Due to the cross-sectional character of the study, it was not possible to collect kidney markers over a period of three months as normally required for a final CKD diagnosis [18]. Instead, we collected kidney markers once during the study. However, most studies investigating CKD prevalence also use a one-time-only evaluation of GFR, e.g., the US Renal Data System Report in 2012 [31].

Our study population was not representative of the general adult Malawian population. Since the study site was urban HTC centre, we recruited more HIV-positive individuals compared to the national prevalence of HIV. This might have also led to a bias among HIV-negative participants, since individuals who felt sick, suspected that they had an HIV-infection and sought testing at the HTC, thus resulting in more patients who were sick, but still HIV-negative than in the general population.

Lastly the cross-sectional character of the study did not allow for a prognosis of whether or not the renal function of patients who were HIV-positive would improve with ART [32].

Although the CKD prevalence of our study-population was lower than in the aforementioned Blantyre study performed in 2009, the results of this study underline the public health importance of CKD. The prevalence of impaired renal function seems to be higher in HIV-positive individuals even though the most advanced stages of reduced eGFR occurred in two HIV-negative individuals in our study. Despite the small number the finding could reflect the problem of late presentation and the lack of diagnosis of renal impairment in the Malawian health care system.

A particular strength of this study was that it also examined eGFR in HIV-negative individuals who are less frequently described in scientific literature concerning CKD in SSA compared to HIV-positive patients.

## Conclusion

Our study confirms that there is a higher prevalence of CKD in HIV-positive individuals in Malawi, however the rate is less pronounced than previously reported in a 2009 study. The limited resources in countries like Malawi preclude general renal screening even in HIV-positive patients prior to the initiation of ART. Therefore, an easy-to-use risk score for targeted screening would be desirable to identify and monitor the renal function of patients with a high risk for CKD. Finally, longitudinal data from settings like Malawi with a differentiated assessment of the type of renal disease are urgently needed.

## Additional file

Additional file 1: Dataset. (XLSX 62.2 kb)

## Abbreviations

ACR: Albumin-creatinine ratio
ART: Antiretroviral treatment
BMI: Body mass index
BP: Blood pressure
CKD: Chronic kidney disease
eGFR: Estimated glomerular filtration rate
ELISA: Enzyme-linked immunosorbent assay
HIV: Human immunodeficiency virus
HIVAN: HIV-associated nephropathy
HTC: HIV testing and counselling centre
IIFT: Indirect immunofluorescent assay
K/DOQI: National Kidney Foundation Kidney Disease Outcome Quality Initiative
LMIC: Low- and middle-income countries
NCD: Non-communicable diseases
SSA: Sub-Saharan Africa
TB: Tuberculosis
TDF: Tenofovir disoproxile fumarate
